# Molecular dynamics simulation of thermal activation of human TRPV1

**DOI:** 10.22099/mbrc.2025.53301.2171

**Published:** 2026

**Authors:** Juan David Bermudes-Contreras, Luis Manuel Arratia-Cortés, Maria Esther Ramírez-Moreno, Beatriz Zamora-López, Cesar López-Camarillo, Laurence A. Marchat, Absalom Zamorano-Carrillo

**Affiliations:** 1 Sección de Estudios de Posgrado e Investigación, Escuela Nacional de Medicina y Homeopatía, Instituto Politécnico Nacional, Ciudad de México, México; 2 Departamento de Psiquiatría y Salud Mental, Facultad de Medicina, UNAM, Ciudad de México, México; 3 Posgrado en Ciencias Genómicas, Universidad Autónoma de la Ciudad de México, Ciudad de México, México

**Keywords:** TRPV1 channel, Heat activation, Molecular dynamics, Pore

## Abstract

TRPV1 (Transient Receptor Potential Vanilloid 1) is a non-selective ion channel that responds to various thermal, chemical, mechanical, and ligand stimuli. It is expressed in various tissues, mainly in nociceptive neurons, adipocytes, and other cell types. This channel is involved in pain and temperature transition processes, although it has recently been implicated in adipocyte browning processes. That is why the study of this receptor has increased in recent years to understand the process of activation and inactivation by various stimuli. In this work, we focus on modeling a complete channel of human TRPV1 (hTRPV1), the structural changes, and especially the behavior of the pore when this protein is subjected to high temperatures (400 K). We report that when molecular dynamics simulate hTRPV1 at 400 K, it suffers an increase in the diameter of the two gates reported elsewhere in the pore, suggesting the opening of the channel. In this work, we describe the structural changes in the entire protein, concomitant to those in the pore, favoring this process, which might be associated with its biological activity.

## INTRODUCTION

Transient receptor potential (TRP) proteins are a superfamily of nonselective cation channels found in eukaryotic cells that can be activated from a closed to an open state in response to various physical and chemical stimuli, such as heat, cold, voltage, acid, and ligands. TRPs can be divided into seven subfamilies based on amino acid sequence homology: TRPA (Ankyrin), TRPC (Canonical), TRPM (Melastatin), TRPML (Mucolipin), TRPN (NO-mechano-potential, NOMP), TRPP (Polycystin), and TRPV (Vanilloid) [1-3]. The TRPV family includes six members named TPRV1-6, all of which are ion channels participating in temperature sensation, pain perception, and other cellular functions. A key member of this family is TRPV1; this receptor is mainly expressed in nociceptive neurons, mainly in the skin and mouth, where it detects painful stimuli produced by many substances and stimuli such as high temperature. Several studies have demonstrated the participation of TRPV1 in the transduction of nociceptive stimuli and the generation of painful sensations, including cancer, neuropathic, postoperative, and musculoskeletal pain, among others, which makes this channel an interesting therapeutic target for pain management [4, 5]. TRPV1 is highly expressed in adipose tissue, where it is involved in processes related to adipogenesis, browning, and food intake, indicating its potential as a target for developing drugs for obesity control [6]. Finally, it has also been described as expressed in skeletal muscle, smooth muscle, epithelial, and immune cells, where it plays other roles [5]. The functional TRPV1 channel comprises four transmembrane molecules of 839 amino acids whose intracellular N-terminus features six ankyrin repeats that fold into six alpha-helices. Each monomer of TRPV1 is organized into six transmembrane segments (S1-S6), forming both the voltage-sensing domain (S1-S4) and the pore-forming domain (S5-S6). These segments are interconnected by a pre-S1 section that links the TRPV1 monomers. The intracellular C-terminus contains a TRP domain (TRP-D) that interacts with pre-S1, several phosphorylation sites, calmodulin, and phosphatidylinositol-4,5-bisphosphate (PIP2) binding sites, all of which are essential for regulating TRPV1 activity. Transmembrane and intracellular domains flank a membrane-proximal domain (MPD) associated with heat activation. The S3 and S4 segments create the vanilloid pocket, the binding site for ligands such as Capsaicin. A conformational change occurs between S4 and S5 upon ligand binding, leading to the opening of the cation-selective pore and subsequent activation of TRPV1 [7, 8]. In this opening process, two gates appear to prevent ion permeation in the absence of activators, such as the ion selectivity filter on the outer side of the pore and the S6 helices lining the cytolytic half of the pore, where a mutual coupling between these two gates allows ion entry. However, the mechanism by which this occurs has not been fully elucidated. Amino acids within these human and rat gates have been reported as Thr641, Gly643, Gly644, Met645 (Met644 and Gly645, in rat) for the gate on the outer side of the pore and for the inner or cytosolic gate Tyr671, Tyr672, Leu679 (Ile679 in rat), Ile680, Leu681 and Val686, which have been proposed to change their conformation during the process of channel activation, being reported mainly in the presence of ligands. However, their behavior when the channel is subjected to different temperatures has not been described [7, 9-12]. Various crystals of rat TRPV1 channels have been obtained and used as templates for modeling the human channel, considering the high protein homology between these species. They were also utilized in molecular dynamics studies to understand the channel's behavior under different stimuli. The human TRPV1 was recently successfully crystallized but does not correspond to the full-length protein. However, this model is a valuable reference for validating other TRPV1 models [12].

In molecular dynamics studies, simulations of TRPV1 at 30°C reveal that the protein is in a closed state. In contrast, simulations conducted at 60°C and 72°C demonstrate conformational variations, indicating that both the upper and lower gates of the channel open. Additionally, simulations at the highest temperature show conformational changes in the MPD linker domain, the external pore, and the TRP helix. Notable changes were also observed in critical regions, such as the S2-S3 and S4-S5 linkers, during channel activation, along with alterations in hydrogen bonding patterns [13, 14]. Considering the relevance of TRPV1 in pain and obesity, this channel receptor appeared to be an interesting target for the computational design of new ligands. Therefore, it is interesting to describe the conformational changes of the hTRPV1 under different temperature conditions. For this purpose, we constructed a model of the full-length human TRPV1 in its tetrameric form and performed molecular dynamics simulations at 310 K and 400 K. The changes in secondary structure and root mean square fluctuation (RMSF) indicate the conformational movements accompanying the channel opening, which is further supported by an analysis of the channel pore.

## MATERIALS AND METHODS

### Homology modeling of human TRPV1 structure:

The three-dimensional model of the monomeric human TRPV1 (UniProtKB-ID Q8NER1) was predicted by the SWISS-MODEL [15], RAPTOR X [16] and I-TASSER [17] servers using the high-resolution crystallographic structure of the tetrameric rat TRPV1 (PDB-ID 3J5P) as a template for homology modeling, since both proteins share 85.3% identity and 92% similarity. The quality of the three models was assessed by the Ramachandran plot and other parameters obtained by PROCHECK [18] and MolProbity servers [19]; protein structure was optimized by the 3Drefine server [20] and stereochemical quality was checked again by PROCHECK and MolProbity servers to achieve the best model of the monomeric hTRPV1. This structure was used to construct the tetrameric hTRPV1 using the UCSF Chimera X [21] and the coordinates of the rat TRPV1 tetramer (PDB-ID 3J5P) served as a template.

### Molecular dynamic simulation (MDS):

The MDS of hTRPV1 was performed with GROMACS 5.1.4 [22, 23] using the OPLS-AA force field at 310 K and 400 K [24]. We used the all-bond constraints LINCS algorithm and the leap-frog algorithm for integrating Newton’s equations [25]. In each condition, the protein was solved in a rectangular box of Single Point Charge (SPC) water [26]. A minimum distance of 1 nm from each protein to the edge of the box was established, and periodic boundary conditions were also applied. To neutralize the electric charges in the system, counter-ions were added, and water molecules were removed if they overlapped with the ions. During energy minimization, the steepest descent algorithm was used until convergence. Further equilibration of the system was accomplished in 5000 steps (10 ps) of MDS with restricted protein atoms and NVT conditions. MDS were performed with a time step of 2 fs under NPT conditions such that the size of the box fluctuated to maintain the pressure at a constant value. The PME algorithm was used for electrostatic interactions with a cut-off of 1 nm [27]. The van der Waals interactions used 1 nm as a single cut-off. Temperature and pressure coupling were performed with the Nosé-Hoover algorithm [28, 29] and the Parrinello-Rahman algorithm [30, 31], respectively. After stabilization of the system, the potential energy was conserved during MDS. Finally, MDS was performed for 100 ns at 310 K and 400 K to evaluate the effect of temperature on protein structure and obtain more conformations using the same initial conformation. The results were analyzed using tools of the GROMACS package such as root-mean-square-desviation (RMSD), root mean square fluctuation (RMSF), the radius of gyration (Rg), and solvent-accessible-surface-area (SASA). The evolution of secondary structures was also characterized throughout the simulation time. Finally, RMSD values were used to perform clusterization and select the most representative 3D structure in each simulation condition. Its quality was verified by the Ramachandran plot obtained from the PROCHECK software, and its graphical representation was obtained using PyMol and VMD programs [32, 33].

### Pore analysis:

 Representative images of the dynamics at 310 K and 400 K were selected by clustering the models. These representative models were used on the server MoleOnline to determine the channel pore size [34].

## RESULTS

As mentioned, TRPV1 plays an important role in the transduction of pain signals, particularly in neuropathic [4]. Its role in adipose tissue makes it an interesting target for treating obesity by allowing greater metabolic activity in adipocytes, decreasing their population [6]. Therefore, the development of drugs that regulate its activity has been a strategy for the control of obesity-related diseases. However, the reduction of unwanted effects, such as hyperthermia, hypothermia, and desensitization, after the administration of these drugs is still a challenge. Therefore, using bioinformatics tools for drug development has become a critical pathway for minimizing these effects. Thus, obtaining TRPV1 structures under different conditions that allow its study in active and inactive forms facilitates a more logical and rational approach to developing new therapies for this target [35]. Therefore, this work provides structural information on TRPV1 that can be used to design new therapies. 

The 3D model of the full-length human TRPV1 is presented in this work. Until now, this is the first report to describe the conformational changes of the full-length human tetrameric form of the channel through molecular dynamics. This computational exploration was performed at two different temperature conditions (310 K and 400 K). Although the crystallographic structure of TRPV1 has been reported, it is worth investigating the phase space of the conformations by molecular dynamics to gain insight into its operation under thermal stress.

**Figure 1:**
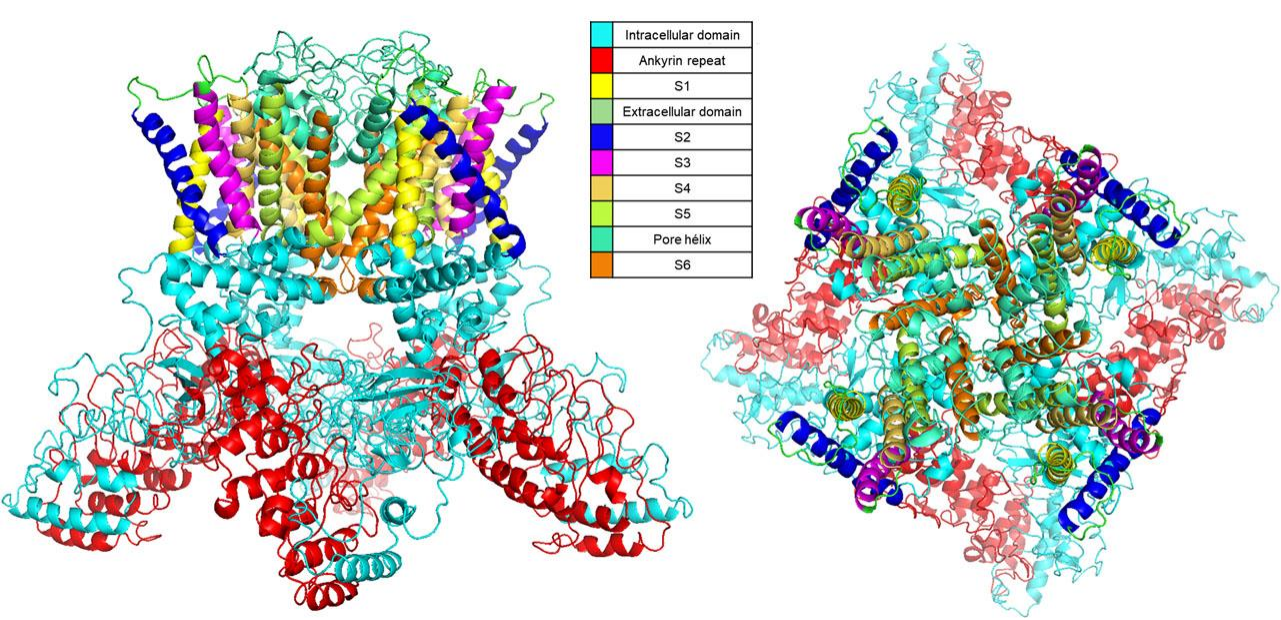
Three-dimensional structure of TRPV1 human protein. A) Side view and B) top view of the tetrameric model of hTRPV1. hTRPV1 consists of six transmembrane domains (S1-S6): S1 in yellow (434-454 aa), S2 in blue (472-497 aa), S3 in magenta (511-531aa) and S4 domain in mustard (536-556 aa) that constitute the voltage sensing domain and the S5 in green (572-599 aa), pore helix in aqua green (600-658 aa) and S6 in orange (659-687 aa) form the pore domain, and long intracellular N- and C- terminal tails. Within the N-terminal tail, six ankyrin repeat domains in red (111-359 aa) allow the binding of calmodulin and ATP to modulate hTRPV1 activation.

The three-dimensional model of the monomeric human TRPV1 (UniProtKB-ID Q8NER1) was predicted by the SWISS-MODEL (with a size of 609 amino acids), RAPTOR X (with a size of 774 amino acids) and I-TASSER (with 839 amino acids) servers. The 3D model of tetrameric human TRPV1 (839 aa) obtained by I-TASSER represents the full-length protein and includes all the functional domains reported elsewhere: the voltage sensor domain comprising segments S1-S4, the pore domain consisting of segments S5-S6 and the pore helix, and segments S2-S5 of the vanilloid pocket where most interactions with exogenous ligands occur. The tetramer was constructed from this model, as shown in Figure 1.

The human TRPV1 tetramer model is quite similar to the rat TRPV1 crystal, and the root-mean-square deviation (RMSD) value of 2.61 Å confirms the significant similarity between both structures. Notably, the superposition of both proteins showed they are very similar in the transmembrane region; they are conserved proteins and have an identity of 86%. Moreover, in a structural comparison between the 8GF8 crystal (human TRPV1) and the model obtained, we assessed an RMSD of 2.59 Å, implying a high similitude, however, the two structures differ in that the 8GF8 crystal structure lacks the first 114 amino acids corresponding to the N-terminal end, as well as 26 amino acids corresponding to the pore helix and the last 70 amino acids corresponding to the C-terminal end. (Supplementary Fig. 1). 

**Figure 2:**
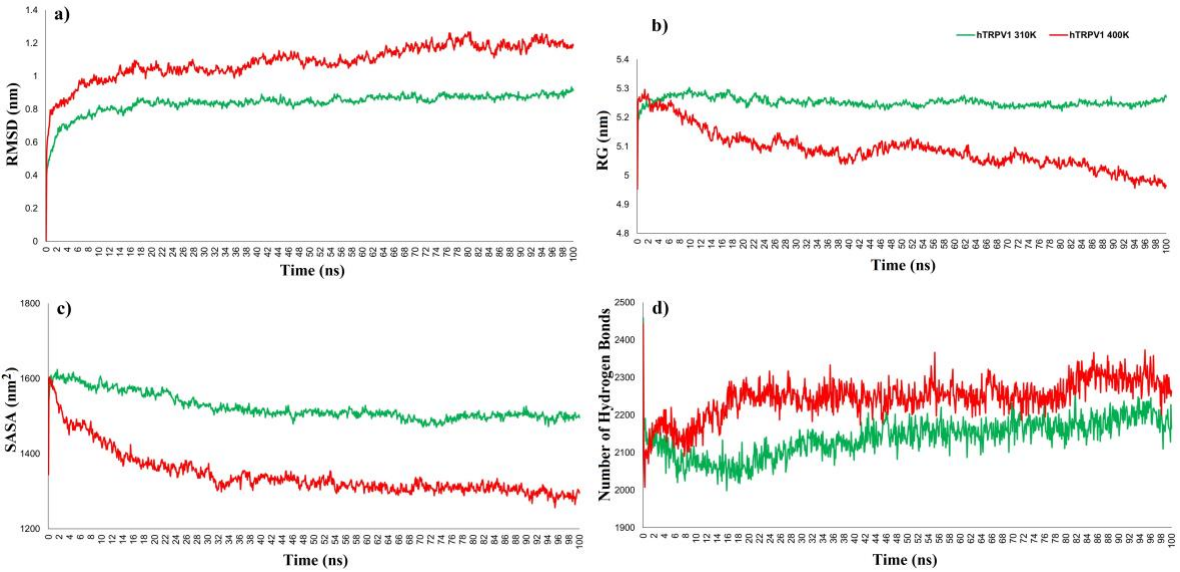
Dynamics of the geometric parameters of hTRPV1 during MD simulation. a) RMSD, b) Radius of gyration, c) SASA, d) H-BONDS.

To explore the temperature-dependent conformational dynamics of hTRPV1, we performed molecular dynamics simulations for the tetrameric form of hTRPV1 during 100 ns. Figure 2a shows the RMSD of TRPV1 at 310 K and 400 K, reaching stability at 20 ns without significant fluctuations in the RMSD values at both temperatures. However, a lower average value of 310 K is obtained (0.8 nm) compared with 400 K (1.2 nm).

**Figure 3:**
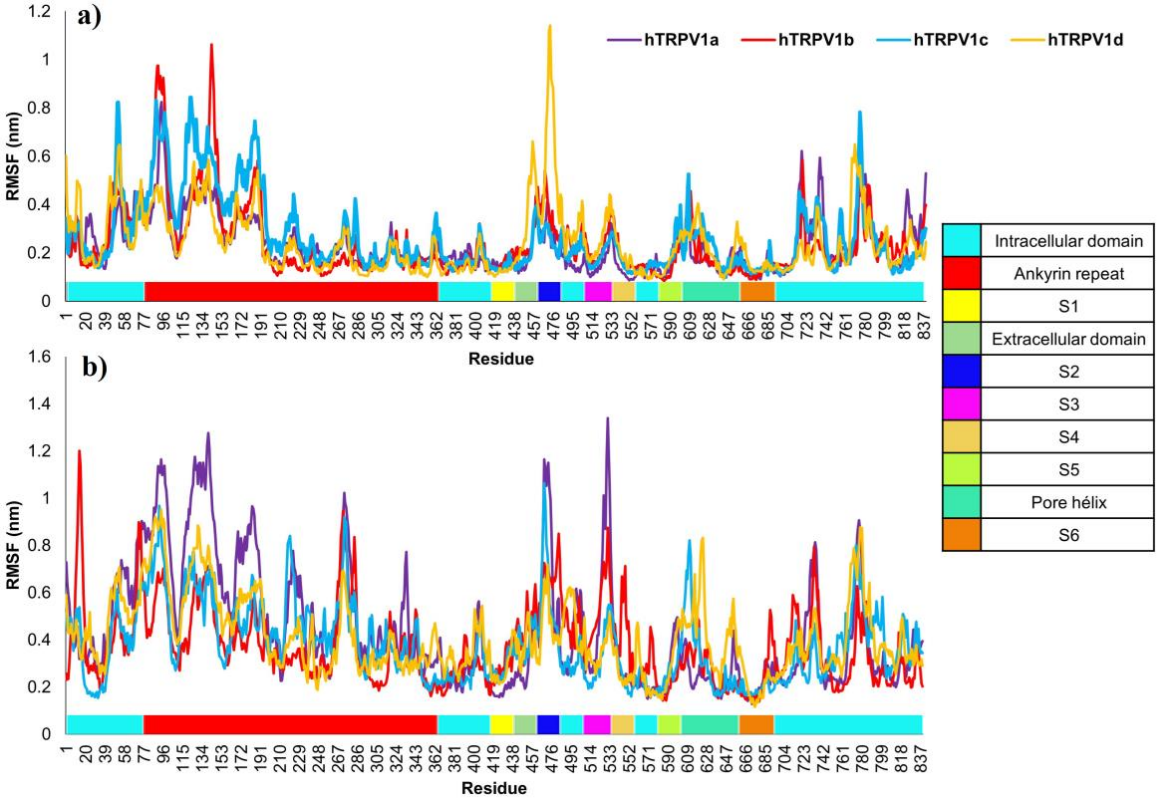
Root mean square fluctuation of human hTRPV1. RMSF at two temperatures: a) 310 K and b) 400 K.

In the gyration radius (Rg) (Fig. 2b), we observed that the dispersion of atoms from the center of mass remains constant at a temperature of 310 K (5.3 nm). In contrast, at 400 K, there is a subtle decrease to 5 nm at the end of the simulation. This movement could be associated with the one that the pore domain (600-658 aa) presents. As observed in Figure 3b, at 400 K, the pore domain tends to move as the voltage sensing domain (S1-S4) to promote the pore opening, while at 310 K, this structure does not suffer variations because the channel remains closed, as seen in Figure 5b. 

SASA calculation (Fig. 2c) shows the opening of holes on the surface of hTRPV1. These holes are measured in surface units and refer to their capacity to hold solvent molecules (water) inside. At 310 K, an average of 1600 nm^2^ was maintained throughout the simulation, while at 400 K, it was reduced to 1400 nm^2^. This reduction of the SASA could be associated with restricting superficial holes in favor of a channel pore. The hydrogen bonds shown in Figure 2d are more abundant in the dynamics at 400 K than at 310 K, suggesting a compaction of the pore walls favoring its diameter. Altogether, the results of Rg, SASA, and H-bonds suggest that hTRPV1 reduced the occupied space due to thermal exposition, and proximity among residues could be the result of the channel opening at 400 K, as observed in Figure 3b, where the amino acids corresponding to the pore have a higher RMSF value.

Figure 3 shows that hTRPV1 has similar RMSF behavior at 310 K and 400 K; the amino and carboxyl ends and ankyrin repeats significantly move. Particularly, S2, S3, and S4 domains, which belong to the voltage sensor domain, also have significant movement. On the other hand, regions forming the pore domain constituted by S5, the pore helix, and S6 also present a pronounced movement that is even more significant at 400 K; at this simulation temperature, the channel pore is expected to open to allow the entry of ions. It is important to highlight the high RMSF values in the ankyrin repeat region since it is located at the amino-terminal end, allowing it to experience greater mobility as it does not interact proximately with other protein subunits. 

**Figure 4:**
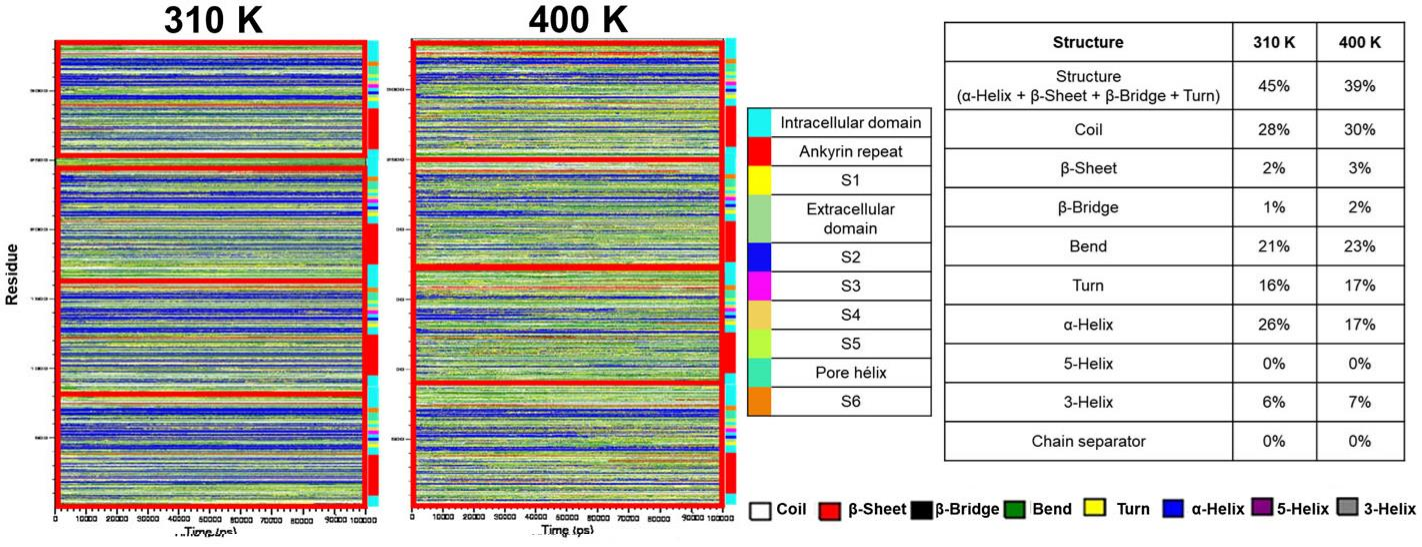
Temporal evolution of the secondary structure of hTRPV1 during MD simulation. Secondary structure at 310 and 400 K. Qualitative (left) and quantitative (right) evolution in the average structure expressed in percentage.

Figure 4 shows the evolution of the secondary structure in the hTRPV1 protein at 310 K and 400 K. A global reduction of the secondary structure was detected (45 to 39%), caused in a greater proportion by a more significant loss of alpha helices comparing the conformations at 310 K versus 400 K (26 to 17%); however, the secondary structure is preserved at both temperatures. In addition, the segment S5-Pore helix-S6 maintains mostly its structure, while S2 loses it, together with the loss of alpha helices in the ankyrin domains. 

**Figure 5:**
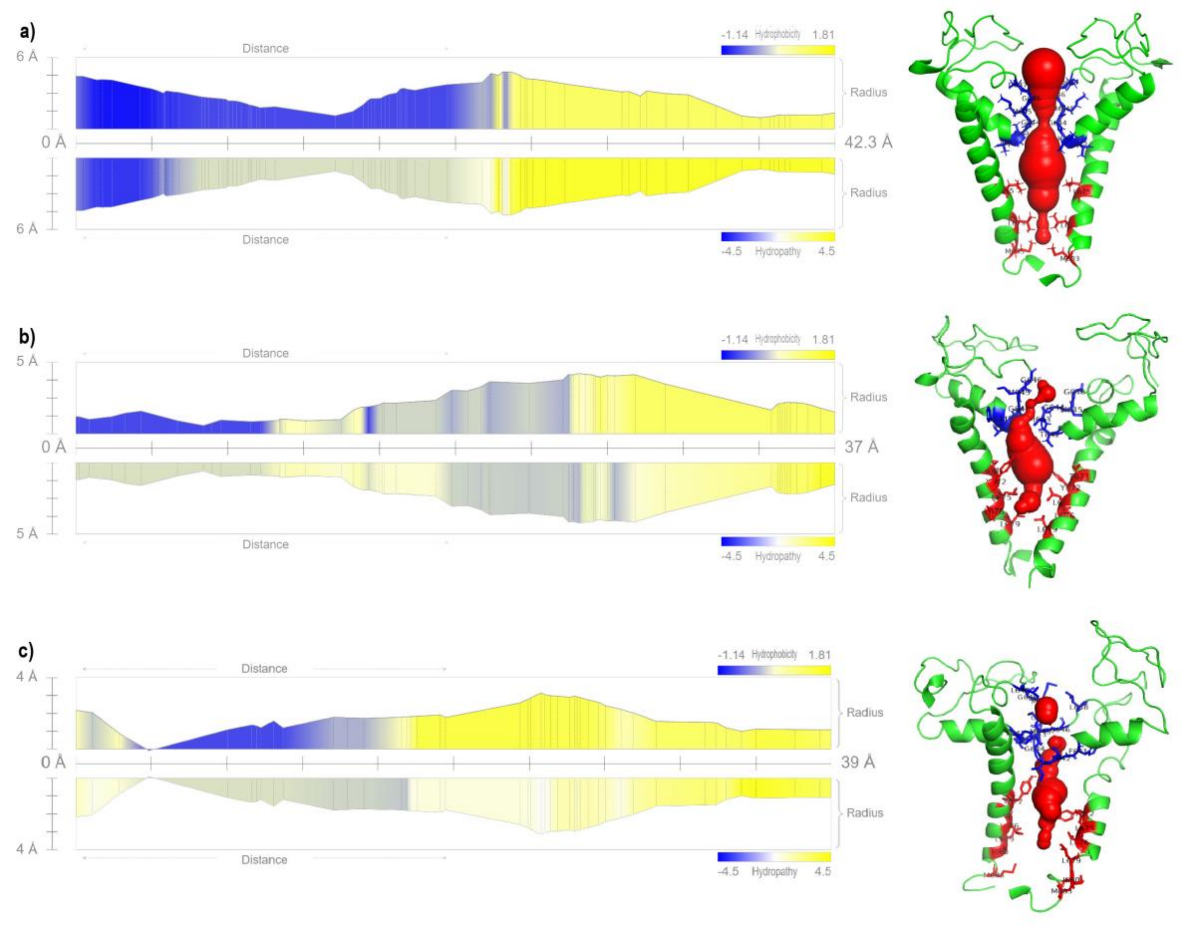
Pore analysis of hTRPV1. a) Initial model. b) Representative model at 310 K. c) Representative model at 400 K.

To complete the description of hTRPV1 dynamics as a calcium channel, we used the MoleOnline server to analyze its geometric dimensions as a pore, i.e., its length through the membrane and width. We also determined the hydropathy and the hydrophobicity inside the pore using the structures of hTRPV1 at time 0 and the most probable conformations in the simulations at 310 K and 400 K (Fig. 5). In the initial structure (t=0) (Fig. 5a), the channel length is about 42.3 Å and the diameter varies along the channel between 1.5 and 4.5 Å. At the same time, the inner half of the pore in the region near the outer part is poorly hydrophobic and the inner section is more hydrophobic. Once hTRPV1 is simulated at 310 K, a reduction in its length (37 Å) is observed with a maximum diameter of 4 Å (in the center of the pore), where the areas with hydrophobicity are maintained (Fig. 5b). In turn, the representative conformation at 400 K presents again a more elongated pore (39 Å) with a maximum diameter of 3 Å (in the center of the pore).

## DISCUSSION

In previous works, the behavior of TRPV1 was described based on incomplete channel models, mainly from rat protein. Molecular dynamics performed at 30°, 60°, and 72°C (during 200 ns) show that regions corresponding to the membrane-proximal domain (MPD), the S1-S2 junction segment, the S2-S3, and the carboxyl terminus exhibit the most significant variations in RMSF values [14]. A similar observation was made in the molecular dynamics of hTRPV1 at 400 K. The authors also reported a contraction of the S2-S3 junction segment and an expansion of the S1-S2 and S4-S5 junction segments [14, 36]. These conformational changes suggest channel activation, indicating that the channel pore is open, allowing calcium ions to enter the cell and generating a signaling cascade. These changes are described by temperature and ligand activation of the channel, the most studied being capsaicin. Analyzing hTRPV1 through molecular dynamics is a valuable step in drug design strategies, coupled with directed molecular docking and pharmacophore modeling for selecting new ligands [37]. Ligand binding sites depend on the activation or inactivation state of the channel [38]. Therefore, the changes in the channel state could affect the vanilloid pocket conformation of the tetrameric hTRPV1 at 310 K and 400 K. The molecular dynamics study of these sites under different temperature conditions could help to identify better ligands from virtual screening. Thus, the inhibition of the channel's activation has been reported to generate an analgesic effect, and the activation could be beneficial for controlling obesity. 

On the other hand, a previous analysis of the pore performed in a TRPV1 of Rattus norvegicus found similar values of radius and hydrophobicity [39]. Herein, we observed that in the initial conformation (Fig. 5a), Ile643, Gly644, and Met645 form the first gate with a radius of 1.1 Å, while in the lower gate, only Leu679 was found with a radius of 1 Å. On the other hand, for the conformation at 310 K (Fig. 5b), a radius of 1 Å is reported in the upper gate where residues Ile643, Gly644, Met645 are found, and in the lower gate Thr671, Tyr672, and Leu679, the latter having a radius of 1.7 Å. Finally, in the conformation at 400 K (Fig. 5c), we found in the upper gate a radius of 1.8 Å with the amino acids Ile643, Gly644, and Met645, while in the lower one, the radius is 1.5 Å Tyr672, Leu679, and Ile680. With the above, we observe in the conformation at 400 K a larger radius in both gates concerning the initial conformation, which reinforces the pore opening event. In comparison, at 310 K, a smaller upper gate is observed than in the initial conformation. This upper gate size of 1-1.5 Å has been reported in other works where TRPV1 is analyzed under antagonist and oxidation conditions while in the presence of agonists such as resiniferatoxin a radius of up to 3 Å has been reported which is similar to that reported in the present work for the channel opening [7, 9, 10, 12]. Another amino acid that could be involved with the opening and closing of the TRPV1, according to distinct analyzed structures, are as follows. At the initial conformation are suggested Thr642, Gly646, Asp647, Leu675, and Met683; at 310 K, Phe641, Thr642, Gly646, Leu675 and Leu676; and at 400 K, Phe641, Thr642, Gly646, Leu648, Leu675, Leu676, and Met683.

Gathering the calculated parameters, the results suggest a dynamical mechanism of the hTRPV1 behavior, allowing it to regulate this channel pore size. At 310 K, hTRPV1 appears to reach stability, presenting a decrease in pore size and a larger radius of gyration (Rg) than at 400 K, suggesting a conformational change towards the closed state. Meanwhile, at 400 K, an increase in pore size and a lower Rg indicate that the probable opening of the channel is concomitant to an approaching among atoms around the pore. Thus, a collective movement of atoms where those adjacent to the pore move toward the center, and the distant possibly move away, inducing a reduction in pore size. This hypothesis could be related to the "iris-like" symmetric scheme of channel opening proposed by Chugunov et al. 2016 [39].

In this work, the atomistical trajectories of the full-length human TRPV1 at two different temperatures showed that the pore size increases when the channel transits from an inactive to an active form. This observation agrees with other reports on proteins of the same superfamily, such as TRPML1 (a calcium ion channel) [34]. Also, conformational changes of the full-length human TRPV1 revealed increased mobility of key segments, such as the voltage domain, pore helix, and pore domain, under heat stress, presenting them as key domains in the mechanistic of this channel. Notably, the hTRPV1 pore showed variations in size and hydropathy, which could help the atomistic movements in TRPV1 that regulate calcium entry into the cell. 

### Acknowledgements:

This work was supported by SIP-IPN, Mexico (grants SIP20210843, SIP20220687, 20230875 and 20241388). LAM, ERM and AZC were supported by EDI and COFAA-IPN, Mexico.

### Conflict of Interest:

 Author has no financial or any non-financial competing interests.

### Authors’ Contribution:

B-C JD: Conceptualization; methodology; formal analysis; writing – original draft; review and editing. A-C LM: Methodology; formal analysis; R-M E: writing – review and editing. Z-L B: review and editing; formal analysis. L-C C review and editing; M-LA: Conceptualization; methodology; formal analysis; writing – original draft; review and editing; resources. Z-C A: Conceptualization; methodology; formal analysis; writing – original draft; review and editing; resources.
