# Supplementary material for: Molecular dynamics simulation of thermal activation of human TRPV1
Source: Mol Biol Res Commun. 2026;15(1):39–48. doi: 10.22099/mbrc.2025.53301.2171 (PMC12673628; doi:10.22099/mbrc.2025.53301.2171)
Supplement: Supplementary file 1 — Figure S1 [file mbrc-15-39-s001.pdf]

Molecular Biology Research Communications 2026;15(1):39-48 DOI:10.22099/mbrc.2025.53301.2171 MBRC

Original Article Open Access

Molecular dynamics simulation of thermal activation of human TRPV1

Juan David Bermudes-Contreras[1], Luis Manuel Arratia-Cortés[1], Maria Esther Ramírez-Moreno[1], Beatriz Zamora-López[2], Cesar López-Camarillo[3], Laurence A. Marchat[1,*], Absalom Zamorano-Carrillo[1,*]

1) Sección de Estudios de Posgrado e Investigación, Escuela Nacional de Medicina y Homeopatía, Instituto Politécnico Nacional, Ciudad de México, México
2) Departamento de Psiquiatría y Salud Mental, Facultad de Medicina, UNAM, Ciudad de México, México
3) Posgrado en Ciencias Genómicas, Universidad Autónoma de la Ciudad de México, Ciudad de México, México

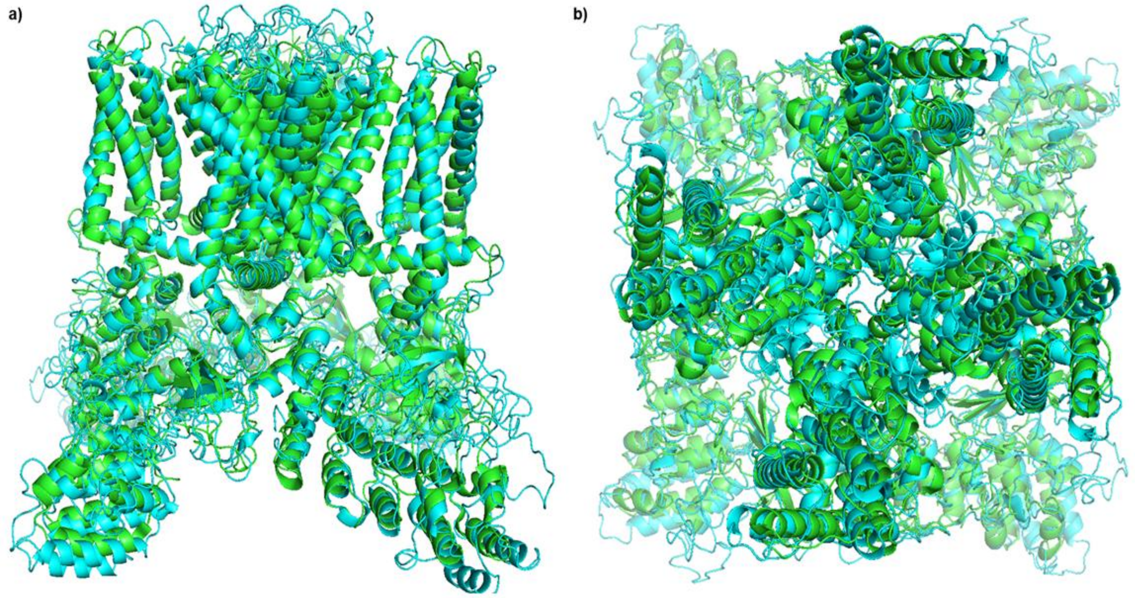

Figure S1: Alignment of the human crystal of TRPV1 (green) with the proposed model in this work (blue). a) Side view and b) top view of TRPV1
